# Introducing Artificial Intelligence Training in Medical Education

**DOI:** 10.2196/16048

**Published:** 2019-12-03

**Authors:** Ketan Paranjape, Michiel Schinkel, Rishi Nannan Panday, Josip Car, Prabath Nanayakkara

**Affiliations:** 1 Amsterdam University Medical Center Amsterdam Netherlands; 2 Department of Internal Medicine Amsterdam University Medical Center Amsterdam Netherlands; 3 Section Acute Medicine, Department of Internal Medicine Vrije Universiteit University Medical Center Amsterdam Netherlands; 4 Lee Kong Chian School of Medicine Nanyang Technological University Singapore Singapore

**Keywords:** algorithm, artificial intelligence, black box, deep learning, machine learning, medical education, continuing education, data sciences, curriculum

## Abstract

Health care is evolving and with it the need to reform medical education. As the practice of medicine enters the age of artificial intelligence (AI), the use of data to improve clinical decision making will grow, pushing the need for skillful medicine-machine interaction. As the rate of medical knowledge grows, technologies such as AI are needed to enable health care professionals to effectively use this knowledge to practice medicine. Medical professionals need to be adequately trained in this new technology, its advantages to improve cost, quality, and access to health care, and its shortfalls such as transparency and liability. AI needs to be seamlessly integrated across different aspects of the curriculum. In this paper, we have addressed the state of medical education at present and have recommended a framework on how to evolve the medical education curriculum to include AI.

## Trends in Health Care

Global health care expenditure has been projected to grow from US $7.7 trillion in 2017 to US $10 trillion in 2022 at a rate of 5.4% [1]. This translates into health care being an average of 9% of gross domestic product among developed countries [2,3]. Some key global trends that have led to this include tax reform and policy changes in the United States that could impact the expansion of health care access and affordability (Affordable Care Act) [4], implications on the United Kingdom’s health care spend based on the decision to leave the European Union [5], population growth and rise in wealth in both China and India [6-8], implementation of socioeconomic policy reform for health care in Russia [9], attempts to make universal health care effective in Argentina [10], massive push for electronic health and telemedicine in Africa [11], and the impact of an unprecedented pace of population aging around the world [12].

From clinicians’ perspective there are many important trends that are affecting the way they deliver care of which the growth in medical information is alarming. It took 50 years for medical information to double in 1950. In 1980, it took 7 years. In 2010, it was 3.5 years and is now projected to double in 73 days by 2020 [13]. This growth is posing a challenge to health care professionals to both retain and use it effectively to practice medicine.

## Rise of Artificial Intelligence in Health Care

### Artificial Intelligence in Health Care

Artificial intelligence (AI) is a scientific discipline that focuses on understanding and creating computer algorithms that can perform tasks that are usually characteristics of humans [14]. AI is now gaining momentum in health care. From its early roots in Turing’s seminal paper, Computing Machinery and Intelligence [15], where he proposed the question “Can machines think?”, AI has come a long way. Examples of advances in AI include natural language processing (NLP) [16], speech recognition [17], virtual agents [18], decision management [19], machine learning [20], deep learning [21], and robotic process automation [22].

Today, AI is being piloted in health care [23] for faster and accurate diagnosis, to augment radiology [24], reduce errors due to human fatigue, decrease medical costs [25], assist and replace dull, repetitive, and labor-intensive tasks [26], minimally invasive surgery [27], and reduce mortality rates [28].

### Challenges With Artificial Intelligence

The rise of AI in health care and its integration into routine clinical practice is going to be a challenge. Along with changing the conventional ways physician work, the *black box problem* [29] and liability issues [30] are some of the most anticipated challenges.

#### Black Box

Researchers at Mount Sinai Hospital have created a deep learning algorithm that was trained on the data of 700,000 patients. This algorithm was able to predict onset of a disease such as schizophrenia with high accuracy [31]. This is even more impressive considering the fact that this condition is difficult to diagnose even for experts. The main problem with this algorithm is that there is no way to know how the system created this prediction and what factors were taken into consideration. This phenomenon is called the *black box* phenomenon. It would not be a precedent in medicine, nevertheless it is difficult to trust a system when there is no understanding on how it works. The physician needs to understand the inputs and the algorithm and interpret the AI-proposed diagnosis to ensure no errors are made. We also need to understand what the consequences or unintended side effects are of black box medicine, even when good outcomes can be demonstrated against a standard of care.

Finally, many of the AI systems attempt to mimic aspects of human and animal central nervous systems that are, at large, still a black box. In a recent paper, Zador [32] argued that we have much more to learn from animal brains to unravel this phenomenon.

#### Privacy and Control Over Data

The development of AI algorithms almost as a rule requires data from a large number of patients. Google, for example, is using 46 billion data points collected from 216,221 adults’ deidentified data over 11 combined years from 2 hospitals to predict the outcomes of hospitalized patients [33,34]. This raises many concerns including relating to patient privacy and control. What happens if a patient does not want to participate in a study where their information is used in algorithm development? In the European Union, the Right to be Forgotten would allow personal data to be erased when the patient has withdrawn their consent [35]. In situations where patient data are limited, algorithm developers train the models on synthetic or hypothetical data, with the risk of generating unsafe and incorrect treatment recommendations [36]. Finally, AI systems are also vulnerable to cybersecurity attacks that could cause the algorithm to misclassify medical information [37].

### Lack of Standards for Use of Artificial Intelligence in Patient Care and Liability

Another unresolved question related to the use of AI in health care is liability for the predictions of an algorithm. It is unclear who is liable when a patient experiences serious harm because of an inaccurate prediction. One could argue for any of the involved parties: the physician, the hospital, the company that developed the software, the person who developed the software, or even the person who delivered the data. Standards for use of AI in health care are still being developed [38,39]. New standards for clinical care, quality, safety, malpractice, and communication guidelines have to be developed to allow for greater use of AI. A recently launched AI system for autonomous detection of diabetic retinopathy carries medical malpractice and liability insurance [40,41].

As use of AI and proactive use of tools such as chatbots [42] increases, physicians and patients will need to be aware of strengths and limitations of such technologies and be trained in how to effectively and safely use them [43,44].

### How Can Artificial Intelligence Address Today’s Physician Challenges?

With medical information growing at a breakneck speed, physicians are having trouble keeping up. This is leading to information overload and creates pressure to memorize all this content to pass the United States Medical Licensing Examinations (USMLE) to qualify for residency positions. Physicians today are working longer hours and are also expected to deliver coordinated care [45,46] in an aging society with complex conditions and comorbidities where health care costs are increasing and regulations are putting an additional burden on administrative processes.

AI could help physicians by amalgamating large amounts of data and complementing their decision-making process to identify diagnosis and recommend treatments. Physicians in turn need the ability to interpret the results and communicate a recommendation to the patient. In addition, AI could have an impact by alleviating the burden from physicians for performing day-to-day tasks [47]. Speech recognition could help with replacing the use of keyboards to enter and retrieve information [48]. Decision management can help with sifting enormous amounts of data and enable the physician to make an informed and meaningful decision [49,50]. Automation tools can help with managing regulatory requirements such as Protecting Access to Medicare Act and enable physicians to review the appropriate criteria before making a cost decision [51]. Finally, to help with the acute shortage of health care professionals, virtual agents could, in the future, help with some aspects of patient care and become a trusted source of information for patients [52].

## Artificial Intelligence Training in Medical Education

### State of Medical Education Today

Physicians go through extensive periods of training before they can eventually register as specialists. Although medicine has seen major changes over the last decades, medical education is still largely based on traditional curricula [53]. The specific length of training differs between countries, but the core competencies of these curricula are globally similar [54]. After a core phase of preclinical didactics, training is mostly centered around practice-based learning [53]. Medical education is often based on 6 domains: patient care, medical knowledge, interpersonal and communication skills, practice-based learning and improvement, professionalism, and systems-based practice [55]. These fields were introduced by the Accreditation Council for Graduating Medical Education (ACGME). A large part of medical training focuses on consuming as much information as possible and learning how to apply this knowledge to patient care. This process is still largely memorization based [56]. Less time is spent on familiarizing medical students or residents with new technologies such as AI, mobile health care applications, and telemedicine [53,55,56]. In the United States, USMLE does not test on these subjects [57]. However, change seems inevitable since the 2018 annual meeting of the American Medical Association (AMA) saw the adoption of AMA’s first policy on augmented intelligence, encouraging research into how AI should be addressed in medical education [58]. In Table 1, several initiatives for incorporating AI in medical education are shown, as presented by the AMA [58].

**Table 1:** Initiatives for artificial intelligence in medical education.

Institution	Project
Duke Institute for Health Innovation	Medical students work together with data experts to develop care-enhanced technologies made for physicians
University of Florida	Radiology residents work with a technology-based company to develop computer-aided detection for mammography
Carle Illinois College of Medicine	Offers a course by a scientist, clinical scientist, and engineer to learn about new technologies
Sharon Lund Medical Intelligence and Innovation Institute	Organizes a summer course on all new technologies in health care, open to medical students
Stanford University Center for Artificial Intelligence in Medicine and Imaging	Involves graduate and postgraduate students in solving heath care problems with the use of machine learning
University of Virginia Center for Engineering in Medicine	Involves medical students in the engineering labs to create innovative ideas in health care

Another important technology-related aspect that is often overlooked in medical training is working with electronic health records (EHRs). EHRs have many benefits, such as improved patient safety, but also assist the implementation of AI in health care. AI algorithms use information from the EHR, and therefore, the knowledge on how to input unbiased data into the EHR is essential. Otherwise, the AI algorithm will likely be biased as well [59]. At present, training on use of EHRs for medical students and physicians is not commonly incorporated in the medical curriculum [60], resulting in the medical professional using the EHR as a replacement to capture information on paper without understanding the true potential of this technology [61]. Training on the use of EHRs usually consists of ad hoc brief introductory courses that just teach the basic skills to use the hospital’s system in practice. Quality of data and concerns on the impact of the computer on the patient-physician relationship are rarely addressed [60] and the USMLE does not test on these subjects either [57].

### How Clinical Practice Is Changing

With the rapid digitization of health care, EHRs facilitate new ways to acquire and process valuable information that can be used to make an informed decision [62]. These advances and transitioning from an information age to the age of AI [56] change clinical practice and patient outcomes for the better. Physicians of the future will have to add to the armory of their skills and competencies, the ability to manage data, supervise AI tools, and use AI applications to make informed decisions.

Physicians will have a crucial role in deciding which of these tools is best for their patients. In turn, this will likely change the physician-patient relationship [63]. When information processing is done mainly by computers, this highlights one of the major benefits of AI in medicine: it allows the physician to focus more on caring for and communicating with patients [64]. Finally, in the age of AI, “the physician should combine narrative, mechanistic and mathematical thinking in their training and consider the biopsycho-social model of the disease with the patient at its center.” “Computers will never substitute for self-reflective medical expert who is aware of the strengths and limitations of human beings and of an environment characterized by information overload” [65,66].

### What Will Be Asked From Physicians in the Future?

Future physicians will need a broad range of skills to adequately use AI in clinical practice. Besides understanding the principles of medicine, physicians will also need to acquire satisfactory knowledge of mathematical concepts, AI fundamentals, data science, and corresponding ethical and legal issues. These skills will help them to use data from a broad array of sources, supervise AI tools, and recognize cases where algorithms might not be as accurate as expected [67]. Furthermore, communication and leadership skills as well as emotional intelligence will be more important than ever as AI-based systems will not be able to consider all the physical and emotional states of the patient [56]. These traits are hard to master for computers and will characterize a great physician in the age of AI.

### Practical Considerations

Some of the time that was originally spent on memorizing medical information will now have to be devoted to other skills. This will have a major impact on the way students and residents will experience their training. The system has to change in such a way that competence will no longer be judged based on factual knowledge but rather on communication skills, emotional intelligence, and knowledge on how to use computers.

With an overfull curriculum, there is limited interest in adopting new topics [68], although a 2016 survey by AMA shows that 85% of physicians perceive benefits from new digital tools [58]. The integration of AI-oriented education into the medical curriculum will take time as the technology evolves. A new infrastructure for learning has to be introduced, and new educators from disciplines such as computer sciences, mathematics, ethnography, and economics will need to be hired. At the moment, these subjects are not even covered by the core competencies of ACGME, but these competencies “are robust enough to adapt to changing knowledge” [69].

To achieve a change in curriculum, many political and bureaucratic hurdles have to be overcome. *Educational systems, program structures,* and *objectives* have to change to create new learning outcomes [70]. A change can only be implemented when large amount of evidence is generated. We have not reached that stage of implementing changes for AI. Furthermore, many other fields within medicine argue that they have not received the attention they deserve [71,72]. AI needs to prove its benefits and also justify that it is an important topic for medical curriculum over other important subjects that lack adequate medical training at present.

However, one of the most compelling arguments for the implementation of AI training in medical education is that this training will augment existing curriculum rather than replace existing coursework. When students are trained to use AI tools, focus should shift from acquiring basic knowledge on how to use the tool to a basic understanding of the underlying principles. This will enable the students to use this fundamental knowledge when current tools get outdated and new tools are introduced.

Another practical problem is that traditional medical training revolves mainly around the interactions between an attending physician and the residents or medical students. When AI is increasingly introduced into clinical practice, this could be problematic. Many senior physicians have little to no experience with AI. AI training could be delivered via Continuing Medical Education (CME) programs and might need to be also taught by educators from outside the medical community. For example, a 2-credit CME course on AI and the Future of Clinical Practice is delivered by a computational biologist and business economists [73].

## Recommendations

### Framework

The traditional medical curriculum, which is mostly memorization based, must follow the transition from the information age to the age of AI. Future physicians have to be taught *competence in the effective integration and utilization of information from a growing array of sources* [56]. To embed this knowledge into medicine, it is of the essence to start introducing these concepts from the beginning of training. In many countries, a Medical College Admission Test (MCAT) has to be taken to be admitted into medical school. The current US MCAT exam, for example, focuses on biology, chemistry, physics, psychology, sociology, and reasoning [78]. These exams could start testing on mathematical concepts such as basis of linear algebra and calculus. These concepts are vital to the elementary understanding of AI and will set the tone for the rest of the curriculum.

In the core phase of preclinical didactics, time should be devoted to working with health data curation and quality [79], provenance [80], integration [81], and governance, working with EHRs [60], AI fundamentals, and ethics and legal issues with AI [82,83]. Course work in critical appraisal and statistical interpretation of AI and robotic technologies is also important [84]. First, these subjects could be taught in self-contained courses to teach about the fundamentals of these subjects that can be used even after current applications become outdated [68]. These self-contained courses could potentially replace and augment courses on medical informatics and statistics in the current curriculum. Second, they should also recur in clinical courses to familiarize students with the clinical applications of AI and work with EHRs in diverse settings [68]. An approach to introducing AI could be to incorporate this technology during courses such as Evidence Based Medicine [85]. As the student is taught to appraise evidence through databases such as PubMed or diagnostic tests or systematic reviews, this process could be augmented by applying concepts from data sciences, applying AI technologies such as NLP and analyzing scenarios to test them on questions of ethics and liability [86]. In addition, the students should also be trained in the fundamentals of computer and software engineering to understand the semantics behind real-world AI applications. For example, basics of hardware and software development and user experience design may also be valuable.

During clinical rotations and residency, focus should shift toward relevant applications of AI in practice. With advancements in digital biomarkers [87] and digital therapeutics [88], students should also be trained in these technologies as they rely on AI. They have the potential to enable large-scale diagnostics and treatments in in-home environments in the near future [89]. At the end of training, the USMLE should include a substantial number of questions on data science and AI fundamentals in their final exams. Attendance of conferences on health care AI could be incentivized, so that health care professionals stay up-to-date with the latest developments. For attending physicians, extensive courses on AI and data science should be part of CME. See Table 2 for more details.

**Table 2:** List of Continuing Medical Education programs on artificial intelligence in health care.

Program	Faculty; organization	Number of Continuing Medical Education credits
Artificial Intelligence and the Future of Clinical Practice [73]	Computational biologist, Business economist; Massachusetts Medical Society	2.0
Intro to AI and Machine Learning: Why All the Buzz [74]	Medical Informatics, Radiology; The Radiological Society of North America	1.0
Current Applications and Future of Cardiology [75]	Healthcare Technologists, Bioinformatics, Cardiology; Mayo Clinic	10.0
Artificial Intelligence and Machine Learning: Application in the Care of Children [76]	Pediatric Medicine; University of Pittsburgh School of Medicine	1.0
Artificial Intelligence in Healthcare: The Hope, The Hype, The Promise, The Peril [77]	Medical Informatics, Business Administration; Stanford University School of Medicine	6.0

AI skills must also be balanced with nonanalytics and person-centered aspects of medicine to develop a more rounded doctor of the future. Other skills such as *communications, empathy, shared decision making, leadership, team building, and creativity are all skills that will continue to gain importance for physicians*. At the Dell Medical School at the University of Texas, Austin, the curriculum in basic sciences has been reduced in duration to accommodate training in soft skills such as leadership, creativity, and communication [63].

To enable clinicians to think innovatively and create technology-enabled care models, multidisciplinary training is needed in implementation science, operations, and clinical informatics. The Stanford medical school has created such a program to train clinician-innovators for the digital future by introducing a human-centered design approach to graduate medical education [90]. At the Healthcare Transformation Laboratory at Massachusetts General Hospital in Boston, a 1-year fellowship is offered in health care innovation exposing resident trainees to topics in data sciences, machine learning, health care operations, services, design thinking, intellectual property, and entrepreneurship [91]. These projects are new developments and are the first steps taken to introduce AI in medical education.

### First Steps

As not all of these interventions can be introduced simultaneously, we suggest a few first steps that will lay the foundation for the upcoming years. We suggest to start off by introducing questions on mathematical concepts into the MCAT similar to the mathematics section in the Graduate Record Examination. High quality Web-based courses on data sciences and AI fundamentals should be freely offered in the core phase of medical education. This might lead to students focusing on applications of these subjects more naturally in following years of training.

For residents and medical students who have already finished this phase of training, courses on the fundamental subjects should be available and mandatory throughout the remaining part of their medical education. For students interested in creating new technology-enabled care models, dedicated training in health care innovation during a gap year during the clinical years or after residency should be encouraged. For attending physicians, introductory courses and refresher courses should also be made available. Extensive training is especially necessary for this group so that they can partly take back the task of educating medical students and residents on these subjects in the future. Table 3 lists suggested content that can be added to the various phases of medical education. Table 4 lists a small subset of rapidly evolving AI in health care conferences that physicians and trainees can attend to learn more about this technology and its applications in health care.

**Table 3:** Recommendations per stage of medical education.

Medical education stage	Recommendations	Suggested content
MCAT^a^	Introduce questions on linear algebra (vectors, linear transformations, and matrix, solutions for linear systems), calculus (limits, differential calculus, and integral calculus), probability (joint, conditional, and distribution)	Education Testing Services’ Graduate Record Examination mathematics test [92]
Medical school—core phase	Working with medical data sets (curation, quality, provenance, integration, and governance), EHRs^b^, AI^c^ fundamentals, and Ethics and Legal	Datasets: HealthData [93] Public datasets in health care [94] University of California San Francisco Data Resources [95] AI fundamentals AI 101 course from MIT^d^ [96] Ethics and Law Teaching AI, Ethics, Law and Policy [97] AI Law [98] EHR training [99]
Medical school—clinical phase	Familiarize with AI-based clinical applications and expand knowledge beyond basic principles of data and AI	Clinical utility: Overview of Clinical applications of AI [100] AI for Health and Health Care (US Department of Health and Human Services) [101] Center for AI in Medicine and Imaging [102] AI in Healthcare Accelerated Program [103]
USMLE^e^	Introduce questions on data sciences, AI, and working with EHRs	Data science courses [104-106]
Residents	Detailed knowledge on clinical applications and attend conference in health care AI	Table 4
Specialist	Stay up-to-date on data/AI through CME^f^ credits and attend conference in health care AI	Tables 2 and 4

^a^MCAT: Medical College Admission Test.

^b^EHR: electronic health record.

^c^AI: artificial intelligence.

^d^MIT: Massachusetts Institute of Technology

^e^USMLE: United States Medical Licensing Examinations

^f^CME: Continuing Medical Education.

**Table 4:** List of artificial intelligence in health care conferences.

Name of conference	Topics
Ai4 AI^a^ Healthcare Conference [107]	Exploring top use cases of AI and ML^b^ in health care
AI in Healthcare [108]	Business value outcomes of AI and experience in clinical care and hospital operations
Machine Learning and AI forum (Healthcare Information and Management Systems Society—HIMSS) [109]	Data, analytics, and real-world applications of ML and AI
AI in Healthcare @ JP Morgan Healthcare Conference [110]	AI applications—drug discovery, secure data exchange, insurer coordination, medical imaging, risk prediction, at-home patient care, and medical billing
Radiology in the age of AI [111]	AI in medical imaging
American Medical Informatics Association Clinical Informatics Conference [112]	AI in medical informatics
Association for the Advancement of AI [113]	“Increase public understanding of AI, improve the teaching and training of AI practitioners, and provide guidance for research planners and funders concerning the importance and potential of current AI developments and future directions”

^a^AI: artificial intelligence.

^b^ML: machine learning.

## Conclusions

Physicians and machines working in combination have the greatest potential to improve clinical decision making and patient health outcomes [114]. AI can curate and process more data such as medical records, genetic reports, pharmacy notes, and environment data and in turn retain, access, and analyze more medical information. However, it cannot replace the art of caring. As AI and its application become mainstream in health care, medical students, residents, fellows, and practicing physicians need to have knowledge of AI, data sciences, EHR fundamentals, and ethics and legal issues concerning AI. Medical schools will need to include them as part of the curriculum. A staged approach to educating the medical student through their journey is recommended.

AI will enable faster and accurate diagnosis, augment radiology, reduce errors due to human fatigue, decrease medical costs, assist and replace dull, repetitive, and labor-intensive tasks, minimally invasive surgery, and reduce mortality rates.

With the global health care expenditure projected to reach US $10 trillion by 2022, AI has the invaluable potential to advance the quadruple aim in health care—enhance the patient experience, improve population health, reduce costs, and improve the provider experience [115,116].

## Abbreviations

AI: artificial intelligence
ACGME: Accreditation Council for Graduating Medical Education
AMA: American Medical Association
CME: Continuing Medical Education
EHR: electronic health record
MCAT: Medical College Admission Test
NLP: natural language processing
USMLE: United States Medical Licensing Examinations
